# Circadian transcriptomic disruptions in the hippocampus precede cognitive deficits in a mouse model of Alzheimer’s disease

**DOI:** 10.4103/NRR.NRR-D-25-00851

**Published:** 2025-12-30

**Authors:** Anlin Qi, Yuxian He, Feng Zhang, Shiyan Liu, Qiuan Xiang, Yanqiong Dong, Bin Wang, Yingying Zhao

**Affiliations:** 1 Department of Physiology, School of Basic Medical Sciences, Health Science Center, Shenzhen University, Shenzhen, Guangdong Province, China; 2 Department of Pathology, The First Affiliated Hospital of Jinan University, Guangzhou, Guangdong Province, China; 3 He Sheng Zhi Li (Shenzhen) Health Technology Co., Ltd., Shenzhen, Guangdong Province, China

**Keywords:** Alzheimer’s disease, circadian rhythm circadian-related genes, cognitive decline, ion homeostasis, neural pathway, neuroinflammation, neuronal function, protein homeostasis, transcriptome analysis

## Abstract

Mounting evidence suggests that circadian rhythm disruption may be linked to the onset and progression of Alzheimer’s disease. However, whether this disruption occurs before the appearance of cognitive symptoms and whether it drives disease development remain unclear. Understanding the temporal relationship between circadian rhythm dysregulation and early Alzheimer’s disease pathological changes may open up new avenues for disease prevention and intervention. To determine if circadian rhythm disruption precedes cognitive decline, we conducted high-resolution transcriptome analyses of the hippocampus in a 5-month-old mouse model of Alzheimer’s disease and age-matched wild-type control mice at multiple time points over a 24-hour period. While the mouse model of Alzheimer’s disease did not exhibit obvious cognitive symptoms at this stage, the expression of circadian-related genes in the hippocampus exhibited extensive abnormalities. In the control group, 2109 genes exhibited rhythmic expression characteristics. In the mouse model of Alzheimer’s disease, a marked proportion of these genes lost their rhythmicity, some genes newly developed rhythmicity, and some maintained rhythmicity but with altered expression patterns. Genes related to neuronal function, including those involved in protein homeostasis regulation, neuroinflammation, and ion homeostasis, showed significant changes in circadian rhythm amplitude and phase, and some completely lost their rhythmicity. These findings point to the following critical early events in Alzheimer’s disease: hippocampal circadian gene disruption occurs before cognitive symptoms emerge, genes related to neuronal function are uniquely susceptible to this early dysregulation, and circadian dysfunction may even precede the pathological changes of Alzheimer’s disease and influence disease onset. This work advances Alzheimer’s disease research by clarifying that circadian disruption is an early pre-symptomatic event, reinforcing the potential of circadian rhythm regulation as a strategy for early intervention of Alzheimer’s disease, and identifying neuronal pathways that may serve as intervention targets.

## Graphical abstract

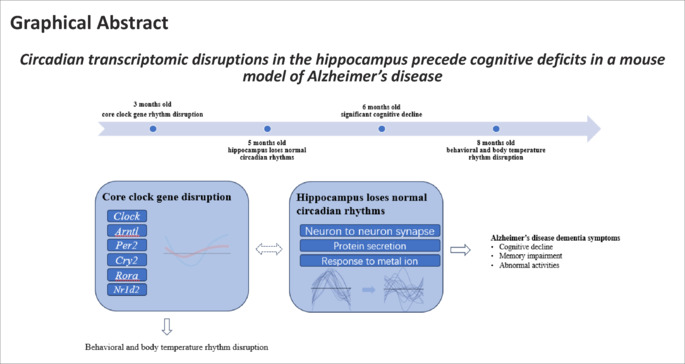

## Introduction

Alzheimer’s disease (AD) is a progressive neurodegenerative disease characterized by cognitive decline, memory loss, and synaptic dysfunction (Leng and Edison, 2021; Tzioras et al., 2023). While these symptoms usually appear in the late stages of the disease, increasing evidence shows that pathological changes such as amyloid-β (Aβ) deposition, tau protein hyperphosphorylation, and neuroinflammation may begin years or even decades prior to symptom onset (Ferrari and Sorbi, 2021; Graff-Radford et al., 2021; Ossenkoppele et al., 2022).

Approximately 70% of patients with early-stage AD experience circadian rhythm disruption and sleep disorders. As dementia symptoms worsen, some patients develop “sundown syndrome” (Clark et al., 2022; Zhang et al., 2022a; Reimus and Sieminski, 2025). Clinical studies have found that AD patients have hormonal imbalances, especially hormones closely related to sleep, such as melatonin, orexin (hypocretin), and glucocorticoids, and appetite-related hormones. The total secretion of these hormones and their 24-hour circadian rhythms are disrupted (Shen et al., 2022). In AD, the harmful buildup of Aβ and tau proteins is often associated with more severe sleep problems, memory loss, and cognitive decline (Nassan and Videnovic, 2022; Rigat et al., 2023; Geng et al., 2025). Recent studies showed that disrupted circadian rhythms may actually exacerbate neurodegeneration and are linked to the progression of AD (Zhao et al., 2017; Yao et al., 2020; Dong et al., 2022). Circadian rhythm disruption may be a preclinical marker of AD and is associated with an increased risk of dementia. This disruption not only reduces the therapeutic effect of treatments targeting the Aβ clearance mechanism but also affects quality of life and may even accelerate disease progression (Nassan and Videnovic, 2022). Therefore, better understanding of the molecular events surrounding circadian rhythm disruption may help identify early biomarkers and establish preventive treatment strategies.

Highlights

• Disruption of hippocampal circadian rhythms in Alzheimer’s disease occurs prior to cognitive symptoms and may drive disease progression.

• A 24-hour high-resolution time-series transcriptomic analysis of the hippocampus reveals new patterns of circadian rhythm dysfunction in neuronal functional genes.

• Abnormal diurnal expression of ion channel genes is present during the pre-symptomatic phase of Alzheimer’s disease.

• Circadian rhythm regulation represents a new target for early intervention in Alzheimer’s disease to slow disease progression.

Circadian rhythms are driven by a network of core clock genes, which coordinate gene expressions over an approximately 24-hour cycle, thereby regulating important physiological and neural functions, including the sleep-wake cycle, synaptic activity, and neuronal metabolism (Colwell, 2021; Keihani et al., 2023; Shen et al., 2023). Circadian rhythm disruption is receiving increased attention in various neurodegenerative diseases, such as Parkinson’s disease and AD.

Whether circadian rhythm disruption is a downstream result of neurodegenerative diseases or an early, independent feature in the pathogenesis of AD remains unknown. Most previous studies evaluated changes in circadian rhythms in AD on the basis of behavioral results or focused on the late stages of the disease, such as focusing on sleep in patients with AD (Zhang et al., 2022a) or after severe cognitive impairment (Knopman et al., 2021), leaving a significant gap in our understanding of the timing and nature of molecular clock dysregulation in early AD. Most preclinical studies have been performed in late-stage AD models. However, new evidence shows that even before the onset of cognitive impairment, the expression of clock genes in hippocampal neurons has changed, leading to rhythmic disorders (Hastings et al., 2023; Morrone et al., 2023; Lee et al., 2024). However, these early-stage studies used low-throughput methods, preventing them from capturing how circadian rhythms are disrupted across the entire genome or identifying which specific biological pathways are affected (Ahmad et al., 2023). Another gap involves the brain tissues and pathways being studied (Takase et al., 2024). Few high-throughput sequencing studies have examined the hippocampus in presymptomatic models of AD, while the hippocampus is a key area for learning and memory and one of the earliest structures affected in AD. Therefore, how the loss of circadian rhythm impacts important processes such as synaptic function, proteostasis, or ion transport is unknown.

In this study, we demonstrate that circadian gene expression in the hippocampus of an AD mouse model was disrupted before the appearance of AD symptoms. Our findings suggest that disrupted circadian rhythms in the hippocampus may serve as an early molecular sign of AD, one that is present even before behavioral symptoms emerge and could potentially contribute to the onset and progression of AD. These results provide a new perspective on how AD unfolds over time and underscore the importance of targeting the circadian rhythm for early diagnosis and intervention strategies.

## Methods

### Animals

Wild-type male C57BL/6J mice were procured from the Guangdong Medical Laboratory Animal Center (Guangzhou, China) with a specific pathogen-free (SPF) grade (license No. SCXK (Yue) 2018-0002, Guangzhou, China). The 5×FAD male mice were sourced from the Jackson Laboratory (Bar Harbor, Maine, USA). Male mice were used to avoid the impact of the estrous cycle in female mice in causing fluctuations in hormone levels, which may affect disease-related indicators. Mice were maintained in specific pathogen-free facilities under a 12-hour light/dark cycle, at 22–25°C, and 40% humidity with unrestricted access to water.

This study was approved by the Institutional Animal Care and Use Committee of Shenzhen University (approval Number: 20190823112; approval date: August 23, 2019). All experimental procedures strictly adhered to the Guide for the Care and Use of Laboratory Animals (Institute of Laboratory Animal Resources (U.S.). Committee on Care and Use of Laboratory Animals), the national standard of the People’s Republic of China GB/T 42011-2022 Laboratory Animals—General Code of Animal Welfare, and the Guiding Opinions on Treating Laboratory Animals Well.

### Groups

This study examined two groups: the 5xFAD group and the control group (wild-type C57BL/6J mice). Mice came from the same breeding colony. For grouping, we used a computer-generated random number table to select mice for each group.

For the behavioral experiments, we analyzed mice at 6, 8, 10, and 12 months of age. We selected 10 mice per group. The sample size for each experiment slightly varied but ranged from 3–10 mice per group; the minimum was 3 mice per group per time point. Details on group numbers are shown in the legends. A total of 29 control mice and 20 AD mice were subjected to behavioral analysis.

For qPCR, we analyzed samples from mice at 3, 5, and 7 months of age. In each age group, we examined subgroups corresponding to six different Zeitgeber Time points (ZT0, ZT4, ZT8, ZT12, ZT16, and ZT20) (*n* = 3 mice/group). A total of 54 control mice and 54 AD mice were analyzed by qPCR.

Hippocampal transcriptome sequencing was conducted on samples from 5-month-old mice. We analyzed four time point groups (ZT0, ZT6, ZT12, and ZT18) (*n* = 3 mice/group). A total of 12 control mice and 12 AD mice were analyzed by transcriptome sequencing.

### Tissue collection

Mice were anesthetized with isoflurane (Baxter Healthcare Corporation, Deerfield, IL, USA) via inhalation using the RWD R500 general-purpose small animal anesthesia machine (RWD Life Science Co., Ltd., Shenzhen, Guangdong Province, China). The anesthesia induction phase was initiated with 4% isoflurane balanced with 100% oxygen (flow rate: 1 L/min), followed by a maintenance phase maintained at 1.5%–2.0% isoflurane (with continued 100% oxygen supply at 0.5 L/min). Depth of anesthesia was confirmed by the absence of withdrawal reflexes to hind paw pinch and stable respiratory rate to avoid incomplete anesthesia or over-anesthesia. Mice were euthanized by cervical dislocation to minimize stress-induced transcriptional changes. Brain tissue was rapidly dissected and immersed in ice-cold phosphate-buffered saline (PBS, Thermo Fisher Scientific Inc., Waltham, MA, USA). Using a sterile dissecting microscope (Carl Zeiss AG, Oberkochen, Baden-Württemberg, Germany) and pre-chilled surgical instruments (Fine Science Tools Inc., Foster City, CA, USA), the two brain regions were sequentially isolated following various anatomical landmarks.

The hypothalamus was dissected, bounded anteriorly by the optic chiasm, caudally by the mammillary bodies, dorsally by the thalamus, and laterally by the internal capsule. Tissue blocks were carefully excised to avoid contamination from adjacent structures such as the hippocampus or cortex. The brain was then placed dorsally upward, and the hemispheres were gently separated along the midline to expose the medial surface, thereby isolating the hippocampus. The hippocampus, located beneath the cerebral cortex, was isolated by lifting its rostral end (near the amygdala) and severing its caudal connection to the entorhinal cortex, ensuring minimal damage while avoiding contamination from the cortex or thalamus. Isolated tissues were immediately snap-frozen in liquid nitrogen (Air Liquide America Corporation, Houston, TX, USA) and stored at –80°C (Thermo Fisher Scientific Inc.).

### Total RNA extraction

Total RNA was isolated from tissues using the FastPure Cell/Tissue Total RNA Isolation Kit V2 (RC112, Vazyme, Nanjing, China), following the manufacturer’s protocol. The concentration and purity of RNA were measured using an Epoch 2 microplate reader (BioTek, Winooski, VT, USA) with a Take3 microvolume plate. Reverse transcription was performed with the HiSlid^TM^ cDNA Synthesis Kit for qPCR (containing dsDNase; Mikx, Shenzhen, China), which was stored at −20°C and thawed on ice before use.

### Real-time polymerase chain reaction

These experiments were performed as previously described (Dong et al., 2022). Real-time polymerase chain reaction (RT-PCR) was performed using cDNA as template with the 2× Polarsignal Color qPCR Mix (Mikx). The mix, stored at –20°C and protected from light to prevent photobleaching of fluorescent components, was thawed on ice and gently mixed by inversion. RT-PCR reactions were performed on a TOptical Gradient Quantitative Thermocycler (Biometra, Göttingen, Germany). The PCR reaction mixture was prepared in 96-well optical reaction plates treated with RNase/DNase-free coatings. Each well contained 10 µL of 2× Polarsignal® Color qPCR Mix (including Taq DNA polymerase, dNTPs, and SYBR Green I dye), 0.5 µL of forward primer (10 µM), 0.5 µL of reverse primer (10 µM), 2 µL of template cDNA (diluted 10-fold as described), and nuclease-free ddH_2_O to adjust the final volume to 20 µL. Each sample was run in triplicate, alongside no-template controls (containing nuclease-free ddH2O instead of cDNA) and reference gene controls (β-actin) on each plate. Plates were sealed with optical adhesive films and briefly centrifuged at 1000 × *g* for 1 minute. The PCR protocol was set as follows: an initial denaturation step at 94°C for 20 seconds, followed by 40 cycles of denaturation at 94°C for 10 seconds and annealing/extension at 60°C for 20 seconds. Fluorescent signals were detected at the end of each extension step. After amplification, a melt curve analysis was performed from 60°C to 95°C (increasing by 0.5°C every 10 seconds) to confirm amplicon specificity. The relative expression levels of target genes were calculated using the 2^–ΔΔCt^ method using β-actin mRNA as the housekeeping gene for normalization. Ct values were analyzed using Microsoft Excel, with triplicate Ct values averaged for each sample. Samples with a coefficient of variation (CV) > 5% among triplicates were re-analyzed. The RT-PCR primer sequences are listed in **Additional Table 1**.

**Additional Table 1:** Real-time PCR primer sequences

Gene	Forward primer (5’-3’)	Product size (bp)
***Bmal1***	F: ACAATG AGC CAG ACAACG	145
	R: TTC CCA TCT ATT GCG TGT	
***Per2***	F: CAC TTG CCT CCG AAA TAA	155
	R: ACT ACT GCC TCT GGA CTG G	
***Cry1***	F: CAC TGG TTC CGAAAG GGA CTC	153
	R: CTG AAG CAAAAA TCG CCA CCT	
***Cry2***	F: CAC TGG TTC CGC AAA GGA CTA	102
	R: CCA CGG GTC GAG GAT GTA GA	

F: Forward; R: reverse.

### RNA-Seq and data analysis

Transcriptome sequencing of hippocampus samples from mice (5 months old) was conducted following the BGI DNBSEQ Eukaryotic Transcriptome resequencing protocol (Naval-Sanchez et al., 2022). Total RNA was extracted from tissues using TRIzol reagent (Invitrogen, Carlsbad, CA, USA), and the quantity and quality were evaluated with a NanoDrop spectrophotometer and an Agilent 2100 bioanalyzer (Thermo Fisher Scientific). mRNA was enriched from total RNA with oligo (dT) magnetic beads to construct libraries, converted to Phi29-mediated DNA nanospheres, and sequenced as 100 bp paired-end reads on the BGIseq500 platform (BGI-Shenzhen, China) (Zhang et al., 2022b). Raw sequencing data were processed with SOAPnuke (v1.6.5) to obtain clean reads saved in FASTQ format (Chen et al., 2018).

The raw reads were subjected to quality control using FastQC (v0.11.9, Babraham Bioinformatics, Cambridge, Cambridgeshire, UK) and low-quality reads were removed using Trimmomatic (v0.39, Usadel Lab, Bielefeld, North Rhine-Westphalia, Germany) with default parameters (Sewe et al., 2022). The resulting clean reads were then aligned to the mouse reference genome (GRCm39/mm39) using the Rsubread (v2.10.0, Liao Laboratory, Walter and Eliza Hall Institute of Medical Research, Melbourne, Victoria, Australia) package for the assembly of unique genes (Liao et al., 2019). Gene expression levels were quantified using the featureCounts function (v2.0.3, CSIRO Bioinformatics, Sydney, New South Wales, Australia) (Liao et al., 2014).

Differential gene expression analysis across groups was performed using DESeq2 (adjusted *P*-value ≤ 0.05) (v1.36.0, Love Laboratory, University of California, Berkeley, Berkeley, CA, USA) (Love et al., 2014). Following the previously published protocol (Whittaker et al., 2023), the JTK_CYCLE algorithm (Hughes et al., 2010) was used to detect periodic characteristics within a 12–24-hour period using a delta value of 6, a period of 24, and the “independent” method, classifying transcripts into rhythmic (JTK algorithm, BH.Q < 0.05) and non-rhythmic (JTK algorithm, BH.Q ≥ 0.05) categories on the basis of a 24-hour oscillation period. The JTK_CYCLE algorithm identified rhythmically changing genes, while the CircaCompare nonlinear cosine regression tool (v1.6.0, Parsons Laboratory, University of Michigan, Ann Arbor, MI, USA) (Parsons et al., 2020) quantified amplitude, Mesor, and acrophase. A rhythmic change between 5xFAD and control groups was inferred when at least one characteristic differed significantly (*P* < 0.05).

KEGG and GO enrichment analyses of the differentially expressed genes were performed using clusterProfiler (v4.4.4, Yu Laboratory, Peking University, Beijing, China) to understand phenotypic changes (BH-adjusted *P*-values < 0.05) (Wu et al., 2021). Visualization of the results was performed using R packages ggplot2 (v3.4.0, Wickham Laboratory, Posit Software, PBC, Boston, MA, USA) (Ginestet, 2011), pheatmap (v1.0.12, Kolde Laboratory, University of Tartu, Tartu, Estonia), and factoextra (v1.0.7, Kassambara Laboratory, Institut de Recherche en Santé, Environnement et Travail, IRSET, Rennes, Brittany, France).

### Monitoring of locomotor activity

These experiments were performed as previously described (Whittaker et al., 2023). The locomotor activity of mice individually housed in PhenoTyper 3000 cages (Noldus Information Technology, Wageningen, the Netherlands) was monitored and analyzed using EthoVision XT V14 tracking software (Noulds Information Technology). These cages have transparent polycarbonate walls, measure 40 cm × 40 cm × 30 cm, contain autoclaved corncob bedding, and have a water bottle attached to the cage wall. Each cage was equipped with infrared sensors that sample at 30 Hz and high-resolution overhead cameras, all to track both the physical movements and spatial positions of the mice. We defined spontaneous locomotor activity as the total distance mice traveled in 3-minute intervals. The software continuously video-tracked the mice for 7 days, with all data saved automatically. The tracking covered both light and dark phases of the light-dark cycle: lights were on from 7:00 am to 7:00 pm and off from 7:00 pm to 7:00 am. The housing room was kept at 23 ± 1°C and 50% ± 5% relative humidity. Before the experiment, the mice underwent a 2-week acclimation period in PhenoTyper 3000 cages under the same light conditions and were handled daily to help them get used to human contact and reduce stress from being touched.

Behavioral recordings were performed by researchers who did not know genotypes to avoid subjective bias. Three core outcome indicators were used to quantify locomotor activity. Distance per hour was calculated by summing the Euclidean distances derived from real-time X/Y coordinate data of the mouse’s center of mass traveled by the mouse within each 60-minute window (unit: cm). The total distance in the dark phase and light phase was obtained by summing the distance per hour values across the entire 12-hour dark phase or 12-hour light phase (unit: cm). The percentage of distance of dark phase and light phase was computed as the total distance of dark/light phase divided by the total 24-hour distance (unit: %).

### Monitoring of core body temperature

This experiment was performed as previously described (Dong et al., 2022). Core body temperature was measured using a rectal probe thermometer (Fisher Scientific International Inc., Hampton, NH, USA). To minimize stress on the mice and ensure accurate measurements, the base of the mouse’s tail was gently held with one hand to minimize movement; the scruff of the neck was not scratched to avoid causing unnecessary stress. Before data recording, a 3-day adaptation period was implemented; mice were handled daily using the same restraint method as during measurement, and the probe was gently inserted into the mouse’s body for a short time (approximately 5 seconds) without recording data. This adaptation period was intended to reduce the anxiety and stress response during subsequent formal measurements. During the experiment, the core body temperature of each mouse was recorded every 4 hours, with three independent measurements performed at each time point; the average value was calculated.

### Novel object recognition tests

This experiment was performed as previously described (Dong et al., 2022). Novel object recognition tests, following established protocols, were conducted to evaluate recognition memory in mice. To minimize stress-induced variability, mice were handled one to two times daily for at least 1 minute for 1 week prior to testing, and on the first day of the experiment, they explored an empty 40 cm × 40 cm × 40 cm square chamber for 5 minutes to acclimatize. During the training session on the subsequent day, two identical objects were symmetrically placed in the chamber for mice to freely explore for 10 minutes. In the 24-hour post-training test, one training object remained while a novel object was placed in the opposite chamber quadrant; mice started from the center and explored for 10 minutes. Exploration time on familiar and novel objects was recorded, defined as whisker sweeping or sniffing within 2.0–3.0 cm. The chamber was cleaned with 75% ethanol after each test. Tests were conducted from 8:00 a.m. to 4:00 p.m., and behaviors were analyzed blindly to reduce bias. The Recognition Index is a core quantitative metric for evaluating recognition memory in the novel object recognition test, designed to quantify the relative preference of mice for the novel object over the familiar one during the 24-hour post-training test session. This index is calculated as the ratio of the total exploration time directed toward the novel object to the sum of exploration times for both the novel and familiar objects, multiplied by 100% (unit: %).

### Morris water maze tests

This experiment was performed as previously described (Dong et al. 2022). The Morris Water Maze tests, initiated at Zeitgeber Time 0 (ZT0) according to an established protocol, were performed to assess spatial learning and memory in the mice. The maze consisted of a circular pool (ZS Dichuang, Beijing, China) with a diameter of 120 cm, filled with water maintained at 22–23°C. To make the water opaque, we added non-toxic white paint. A circular platform, 10 cm in diameter, was submerged 1 cm below the water’s surface. The maze was divided into four quadrants: northwest, northeast, southwest, and southeast. During the tests, a curtain surrounded the pool, and spatial cues were fixed around it. In the acquisition phase, each mouse was given 60 seconds to swim freely and locate the platform. If a mouse successfully found the platform, it remained there for 10 seconds. If it could not find it, we guided the mouse to the platform and also made it stay there for 10 seconds. Each mouse underwent four trials daily, starting from different quadrants, for 5 consecutive days. On the sixth day, the platform was removed. The mice were allowed to swim from the quadrant opposite to where the platform had been located, searching the water for 60 seconds. After each trial, the mice were dried with a towel.

We used the Morris Maze Analysis System (ZS Dichuang) to record and analyze various parameters, including swimming speed, escape latencies (the time taken to find the platform), swimming paths, and the number of times the mouse crossed the target area. The researchers conducting the tests and analyzing the data were blinded to the group assignments. We selected escape latencies from each trial day as the outcome indicator, using the time it took for a mouse to enter the water and reach the hidden platform, with a threshold of greater than 1 second.

### Statistical analysis

Statistical analyses were conducted using the R statistical programming language (R v4.2.2, R Foundation for Statistical Computing, Vienna, Austria) (R Core Team, 2024). Measurement data, such as activity distance, core temperature, escape latency, and gene expression levels, are expressed as the mean ± standard error (SE). The normality of continuous variables was assessed using the Shapiro–Wilk test. For normally distributed data, one-way analysis of variance was used to compare groups with a single independent variable, while two-way analysis of variance was used when there were two independent variables, such as group × age or group × Zeitgeber time. *Post-hoc* pairwise comparisons were performed using the Sidak test for planned comparisons or the Tukey test for all pairwise comparisons. For non-normally distributed data, non-parametric tests were applied, including the one-way Kruskal–Wallis test and the Mann-Whitney *U* test for pairwise comparisons. Statistical significance was defined as *P* < 0.05.

## Results

### Age-related circadian disruption parallels cognitive decline in the mouse model of Alzheimer’s disease

**Figure 1:**
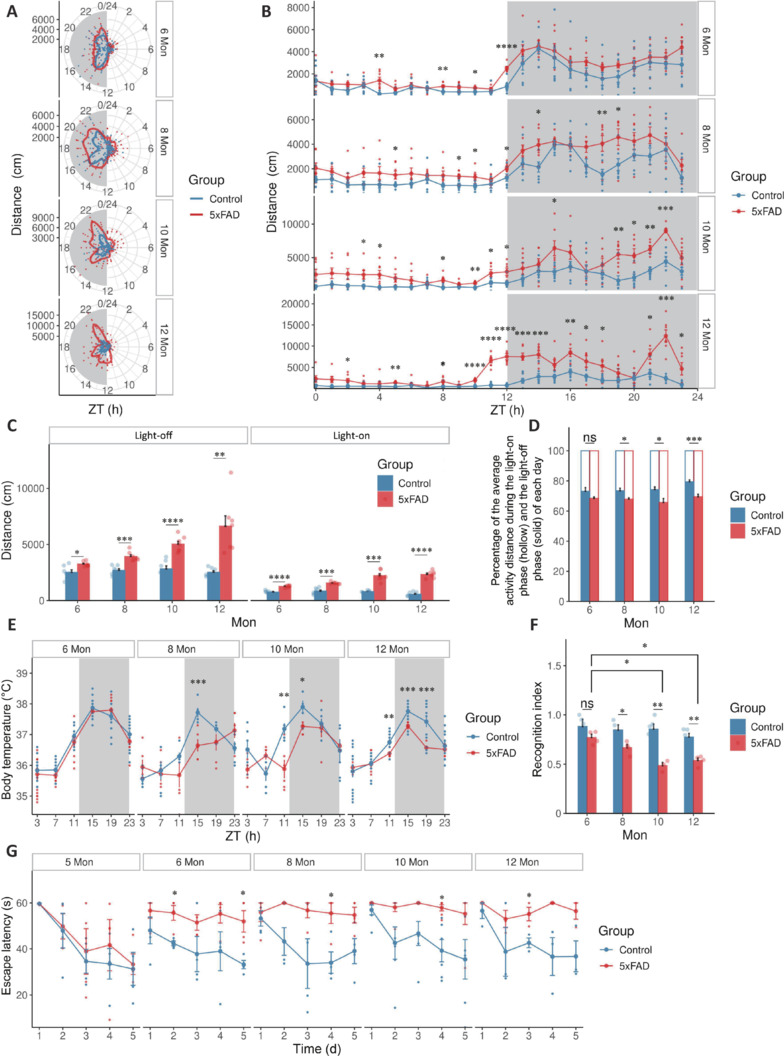
Age-related circadian disruption parallels dementia progression in a mouse model of Alzheimer’s disease. (A, B) Activity distance recordings, as detected by the locomotor activity recorder over 7 days. *n* = 7 per group per age per time point, using one-way analysis of variance followed by Sidak test. (C) The average activity distance during the light-on and light-off stages for 7 consecutive days. *n* = 7 per group per age, using two-way analysis of variance followed by Sidak test. (D) The percentage of the average activity distance during the lights-on phase (hollow) and the lights-off phase (solid) of each day. *n* = 7 per group per age, using two-way analysis of variance followed by Sidak test. (E) 24-hour mouse core temperature recording. *n* = 5-10 per group per age per time point, using one-way analysis of variance followed by Turkey’s *post hoc* test. (F) Recognition index was defined as the time to explore a familiar or new object/total time to explore both objects. *n* = 3–5 per group per age, using two-way analysis of variance followed by Turkey’s *post hoc* test. (G) Escape latency onto a hidden platform during the training trials of the Morris water maze test. *n* = 3–5 per group per age, using one-way analysis of variance followed by Sidak test. Data are represented as the mean ± SEM. *P < 0.05, **P < 0.01, ***P < 0.001, ****P < 0.0001. ns: Not significant; ZT: zeitgeber.

To investigate circadian rhythms in AD model mice, we evaluated the locomotor activity of mice at various ages during light and dark cycles. We used a video monitoring system to continuously track their activity over a 24-hour period. At 6 months of age, 5xFAD mice exhibited a diurnal/nocturnal activity pattern comparable to age-matched wild-type controls, resting during the light phase and being active during the dark phase (**Figure 1A** and **B**). With advancing age, the total 24-hour activity in AD mice markedly increased, accompanied by a progressive disruption of circadian behavioral rhythms (**Figure 1A** and **B**). The 5xFAD mice showed significantly increased locomotor activity both during the day and night compared with wild-type mice (6 months: *P* < 0.05; 8 months: *P* < 0.001; 10 months: *P* < 0.0001; 12 months: *P* < 0.01), with the increase in daytime activity being more pronounced than that during the night (**Figure 1C**). The circadian phase in AD model mice appeared to shift gradually, with the ratio of daytime to nighttime activity increasing over time. Moreover, the percentage of daytime activity became even higher with age (6 months: *P* > 0.05; 8 months: *P* < 0.05; 10 months: *P* < 0.05; 12 months old: *P* < 0.001; **Figure 1D**).

We also assessed core body temperature as another key circadian output. At 6 months of age, the 5xFAD mice maintained a normal circadian temperature rhythm (all time points: *P* > 0.05); similar to the control group, they exhibited higher temperatures during the dark phase and lower temperatures during the light phase (**Figure 1E**). However, by 8 months of age, the AD mice showed significant disruptions in this rhythm (ZT15: *P* < 0.001), which persisted and worsened over time.

To determine whether disturbances in circadian rhythm progress in tandem with cognitive decline, we assessed the recognition and spatial memory of the mice using the novel object recognition test and the Morris water maze. Mice naturally prefer new objects, making the novel object recognition test a measure of recognition memory. At 6 months of age, 5xFAD mice showed no significant impairment in recognition memory compared with controls (*P* > 0.05), but by 8 months, their recognition memory was markedly impaired (*P* < 0.05), with this decline worsening with age (6 months *vs.* 10 months: *P* < 0.05; 6 months *vs*. 12 months: *P* < 0.05; **Figure 1F**). The Morris water maze, which reflects hippocampal function by measuring spatial learning and memory, revealed that 5xFAD mice had no significant deficits at 5 months old (*P* > 0.05); however, hippocampal dysfunction emerged at 6 months (*P* < 0.05; **Figure 1G**). Together, these findings indicate that 5xFAD mice develop noticeable circadian behavioral disruptions as early as 6 months old, coinciding with the onset of hippocampal dysfunction.

### Circadian gene disruption precedes cognitive decline in the mouse model of Alzheimer’s disease

**Figure 2:**
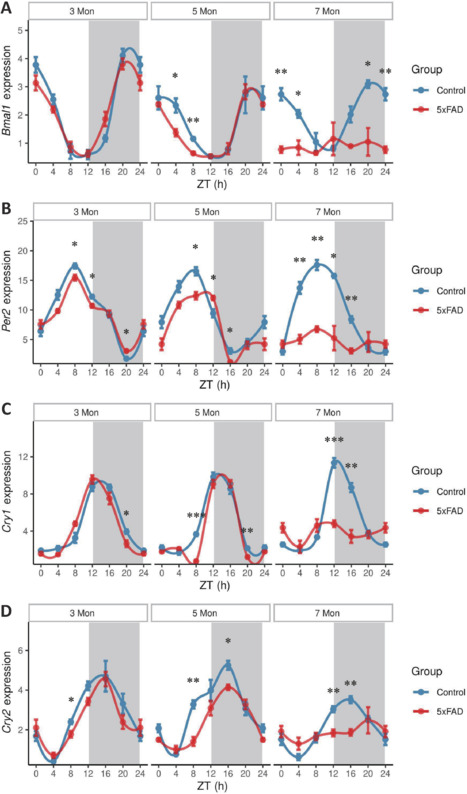
Circadian disruption precedes dementia in Alzheimer’s disease. (A–D) Relative expression level of circadian rhythm–related genes in the hypothalamus at six time points throughout the day, data from ZT0 were reused to represent the ZT24 time point. *n* = 3 per age per time point and per group. Statistical significance represents the comparison between the Control and 5xFAD groups, using one-way analysis of variance followed by Sidak test. *P < 0.05, **P < 0.01, ***P < 0.001. Bmal1: Basic helix-loop-helix ARNT like 1; Cry1: cryptochrome circadian regulator 1; Cry2: cryptochrome circadian regulator 2; Per2: period circadian regulator 2; ZT: zeitgeber.

Our results showed age-related disruptions of circadian rhythms in AD mice. To further investigate the timing of this disruption, we examined the expression of core clock genes, *Bmal1*, *Per2*, *Cry1*, and *Cry2*, in the hypothalamic central clock. Compared with age-matched controls, 3-month-old AD mice maintained similar clock gene expression patterns. At 5 months, Per2 and Cry2 showed altered amplitude and phase. At 7 months, the circadian expression patterns of *Bmal1*, *Per2*, *Cry1*, and *Cry2* were markedly disrupted. (**Figure 2A–D**) These results suggest that central clock dysfunction begins as early as 3 months in AD model mice, consistent with clinical observations that circadian disruption often precedes the onset of dementia symptoms in patients.

### Chronotranscriptomics reveals early circadian gene expression disruptions in the hippocampus of the mouse model of Alzheimer’s disease

**Figure 3:**
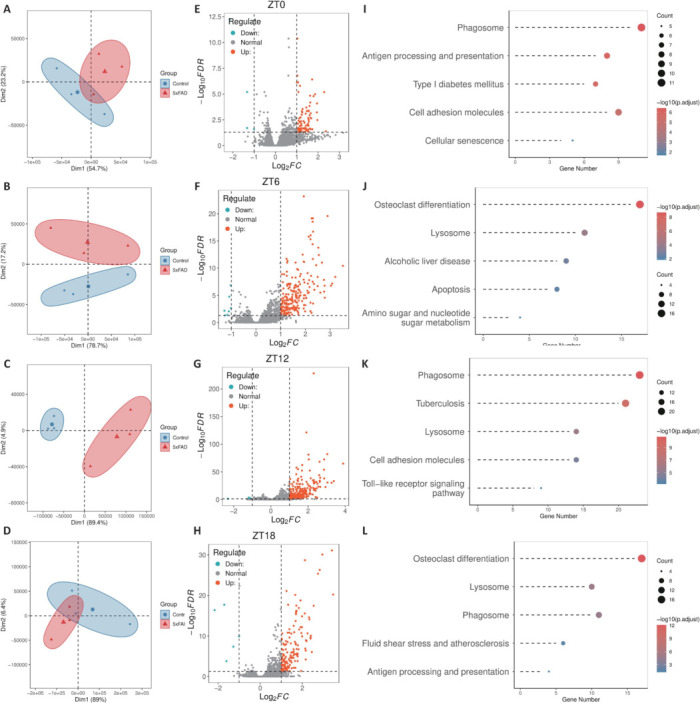
Chronotranscriptomics reveal early circadian gene expression disruptions in the Alzheimer’s disease hippocampus. (A–D) Principal component analysis of the differences in mouse gene expression data at ZT0, ZT6, ZT12, and ZT18 between the 5xFAD and control groups. (E–H) Volcano maps comparing differentially expressed genes between the control and 5xFAD groups and between the ZT0, ZT6, ZT12 and ZT18 data; upregulated and downregulated genes are shown as red and blue dots, respectively, and gray dots indicate genes that are not significantly different (*P*-adjust < 0.05). (I–L) KEGG pathway annotation lollipop plot depicting the genes that show differential expression at the ZT0, ZT6, ZT12 and ZT18 time points between control and 5xFAD groups. KEGG: Kyoto Encyclopedia of Genes and Genomes; ZT: zeitgeber.

At 5 months of age, AD mice exhibit significant alterations in the central circadian clock located in the hypothalamus. To more closely explore whether changes in diurnal gene expression rhythms in the hippocampus occur prior to the onset of cognitive symptoms and examine the potential impact of circadian disruption on dementia, we conducted time-series transcriptomic profiling of the hippocampus in 5-month-old AD mice and age-matched controls across multiple time points (ZT0, ZT6, ZT12, and ZT18) over a 24-hour period. Principal component analysis revealed significant clustering of the two groups at all four time points (**Figure 3A–D**). Differential expression analysis identified 95, 227, 281, and 254 differentially expressed genes (DEGs) in the AD group at ZT0, ZT6, ZT12, and ZT18, respectively (**Figure 3E–H**, adjusted *P*-value ≤ 0.05). DEGs at ZT0 were primarily enriched in pathways related to phagosome, antigen processing and presentation, type 1 diabetes mellitus, cell adhesion molecules, and cellular senescence (**Figure 3I**). DEGs at ZT6 were enriched in osteoclast differentiation, lysosome, alcoholic liver disease, apoptosis, and sugar metabolism (**Figure 3J**). DEGs at ZT12 were mainly associated with phagosome, tuberculosis, lysosome, cell adhesion molecules, and Toll-like receptor signaling pathways (**Figure 3K**). DEGs at ZT18 were enriched in osteoclast differentiation, lysosome, phagosome, fluid shear stress and atherosclerosis, and antigen processing and presentation (**Figure 3L**). Collectively, these findings indicate that 5-month-old AD mice exhibit distinct hippocampal transcriptomic profiles compared with age-matched controls across all tested circadian time points (ZT0, ZT6, ZT12, ZT18). The DEGs were enriched in AD-relevant pathways such as phagosome, lysosome, and antigen processing and presentation, supporting an association between early hippocampal circadian gene expression disruption and AD (Liu et al., 2021; Wang et al., 2025).

### The hippocampus of the mouse model of Alzheimer’s disease at 5 months old loses normal circadian rhythms

**Figure 4:**
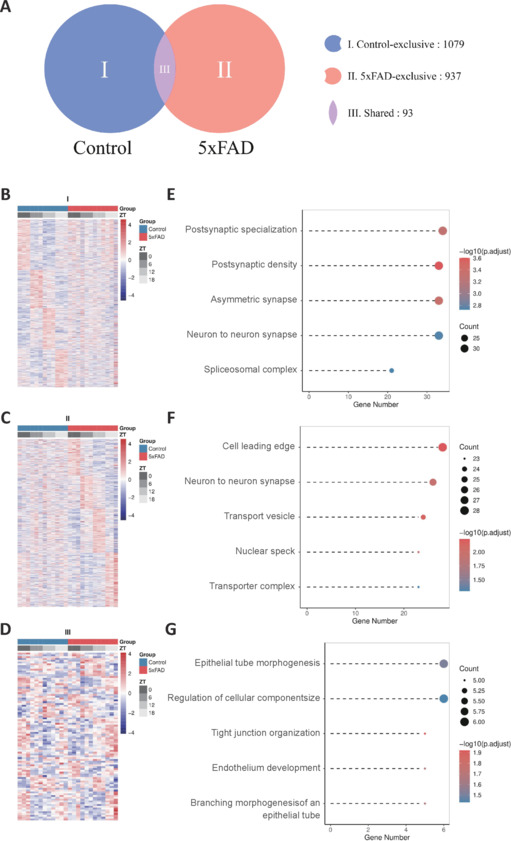
The hippocampus of 5-month-old mouse models of Alzheimer’s disease loses normal circadian rhythms. (A) Venn diagram displaying the number of oscillating genes in hippocampus samples isolated from 5xFAD and control groups. (B–D) Heat maps showing the differentially expressed genes in Part I, II, and III between control and 5xFAD groups. The expression level is expressed in the heat map as the Z-score. Each sample is represented by a column, and each gene is represented by a line. (E–G) Gene ontology functional annotation lollipop plot depicting the genes that show differential expression in Parts I, II, and III between control and 5xFAD groups.

To further characterize changes in hippocampal circadian gene expression patterns in 5-month-old AD mice and explore how the rhythmicity of these genes differs from that in age-matched controls, we analyzed 24-hour transcriptomic data using specialized circadian analysis tools. We used CircaCompare (Parsons et al., 2020) to identify genes exhibiting rhythmic expression, yielding a total of 2109 circadian genes for further analysis (*P* < 0.05). These genes were categorized into three groups: Group I, 1079 genes that exhibit circadian rhythms in the control hippocampus but lose rhythmicity in the AD hippocampus (**Figure 4A** and **B**); group II, 937 genes that gain circadian rhythmicity in the AD hippocampus, despite being arrhythmic in controls (**Figure 4A** and **C**); and group III, 93 genes that retain circadian rhythmicity in both control and AD hippocampi (**Figure 4A** and **D**).

GO analysis of the 1079 Group I genes revealed significant enrichment in pathways related to neurons and synapses, including postsynaptic specialization, postsynaptic density, asymmetric synapse, and neuron-to-neuron synapse (**Figure 4E**). The 937 genes in Group II, which gained rhythmicity in the AD hippocampus, were enriched in pathways associated with the cell leading edge, neuronal synapses, and transport vesicles (**Figure 4F**). Although the 93 genes in Group III maintained rhythmicity in both groups, the oscillation patterns of most genes were altered in AD mice. These genes were primarily involved in pathways related to cell morphology and tight junctions between cells (**Figure 4G**). These enrichment results are provided in **Additional Table 2**. Together, these results demonstrate that 5-month-old AD mice exhibit distinct alterations in hippocampal circadian gene rhythmicity compared with controls, with some genes losing rhythm and others gaining rhythm and retained rhythmic genes showing altered oscillation patterns. Genes involved in neuronal synapses, cell structure, and transport were prominently affected in the hippocampus of AD model mice.

**Additional Table 2:** GO enrichment analysis results

ID	Rich factor	Fold enrichment	*P*_adjust_	Gene ID
**Part I GO Enrichment**				
**GO:0099572**	0.078	2.442	0.001	*Exoc4/Mpdz/Ctnnb1/Prkcz/Shank3/Stat3/Hspa8/Hnrnph1/Dlgap3/Dvl1/Hspb1/Pura/Myo6/Grip1/Pmp/Slc3 0a1/Add3/Pak2/Actn2/Cacng5/Arfgap1/Eef2k/Sema4f/Prr7/Adgrb1/Palm/Camk2n1/Grk3/Chrnb1/Grk2/Cabp1/Sh2d5/Tsc1/Mpp2*
**GO:0014069**	0.086	2.693	0.000	*Exoc4/Mpdz/Ctnnb1/Prkcz/Shank3/Stat3/Hspa8/Hnrnph1/Dlgap3/Dvl1/Hspb1/Pura/Myo6/Grip1/Prnp/Slc30a1/Add3/Pak2/Actn2/Cacng5/Arfgap1/Eef2k/Sema4f/Prr7/Adgrb1/Palm/Camk2n1/Grk3/Grk2/Cabp1/Sh2d5/Tsc1/Mpp2*
**GO:0032279**	0.081	2.540	0.000	*Exoc4/Mpdz/Ctnnb1/Prkcz/Shank3/Stat3/Hspa8/Hnrnph1/Dlgap3/Dvl1/Hspb1/Pura/Myo6/Grip1/Prnp/Slc30a1/Add3/Pak2/Actn2/Cacng5/Arfgap1/Eef2k/Sema4f/Prr7/Adgrb1/Palm/Camk2n1/Grk3/Grk2/Cabp1/Sh2d5/Tsc1/Mpp2*
**GO:0098984**	0.074	2.307	0.002	*Exoc4/Mpdz/Ctnnb1/Prkcz/Shank3/Stat3/Hspa8/Hnrnph1/Dlgap3/Dvl1/Hspb1/Pura/Myo6/Grip1/Prnp/Slc30a1/Add3/Pak2/Actn2/Cacng5/Arfgap1/Eef2k/Sema4f/Prr7/Adgrb1/Palm/Camk2n1/Grk3/Grk2/Cabp1/Sh2d5/Tsc1/Mpp2*
**GO:0005681**	0.094	2.936	0.002	*Aqr/Ctnnbl1/Hspa8/Hnrnph1/Hnrnpr/Cdc40/Zmat5/Snrnp70/Plrg1/Cirbp/Hnrnpk/Wdr83/Luc7l2/Rbm8a/Srrm2/Aar2/Sf3b1/Hnrnpu/Prpf38b/Prpf4b/Ddx23*
**Part II GO Enrichment**				
**GO:0031252**	0.067	2.526	0.006	*Dst/Eps8l1/Trpv2/Tnfrsf12a/Cspg4/Srgap2/Cd44/Arhgap31/Wasl/Psd/Mtmr14/Itgb1bp1/Stx3/Acap2/Inpp5j/Trpm7/Kcnc1/Epb41l5fW'wc1/Kcnh1/Oprd1/Dynlt1a/Cobl/Hcn2/Dlc1/Ablim1/Atp2b2/Pak1*
**GO:0098984**	0.058	2.204	0.012	*Dst/Syn3/Unc13b/Cadm1/Rpl10a/Srgap2/Rab8a/Mtmr2/Sema4b/Rnf19a/Psd/Stxbp5/Cpeb1/Homer3/Syt9/Rgs*7*bp/Magi2/Rtn4/Slc4a8/Lrrtm2/Oprd1/Nr3c1/Dnajb1/Ablim1/Atp2b2/Pak1*
**GO:0030133**	0.065	2.472	0.007	*Syn3/Unc13b/Bloc1s5/Sv2a/Slc40a1/Cadm1/Sec13/Slc2a8/Aph1a/Rab8a/Mtmr2/Lin7c/Bcl2l1/Stxbp5/Syt3/Crispld2/Stx3/Trpm7/Snap29/Syt9/Rph3a/Slc4a8/Oprd1/Syt2*
**GO:0016607**	0.062	2.356	0.012	*Cdk12/Phf7/Pskh1/Ogg1/Fibp/Mecom/Phf5a/Rbm39/Npm1/Zbtb16/Klf15/Aagab/Csnk1a1/Ppp1r16b/Hif3a/Virma/Ehmt2/Nampt/Slu7/S100pbp/Nr3c1/Alkbh5/Rsrc1*
**GO:1990351**	0.051	1.915	0.050	Kcmp3/Hcn4/Kcna3/Pkd1l3/Timm9/Htr3a/Slc26a6/Hspa2/Stxbp5/Ostm1/Lrrc8c/Lrrc38/Scn4b/Kcnc1/Lrrc55/Vwc2/Kcnip4/Kcnh1/Ndufa7/Kcnh7/Hcn2/Cacna1g/Kcna6
**Part III GO Enrichment**				
**GO:0060562**	0.015	5.854	0.031	Ednl/Flt1/Mmp14/Kdr/Brpf1/Etv4
**GO:0032535**	0.014	5.572	0.037	Ednl/Aloxl5/Eif4g2/Ist1/Pecam1/Slc12a6
**GO:0120193**	0.049	18.892	0.012	Cldn10/Pecam1/Cdh5/Cldn5/Tgfb3
**GO:0003158**	0.036	13.764	0.022	Edn1/Pecam1/Cdh5/Kdr/Cldn5
**GO:0048754**	0.026	10.089	0.022	Edn1/Flt1/Mmp14/Kdr/Etv4

### Hippocampal rhythmic genes lose their circadian expression patterns in the mouse model of Alzheimer’s disease

**Figure 5:**
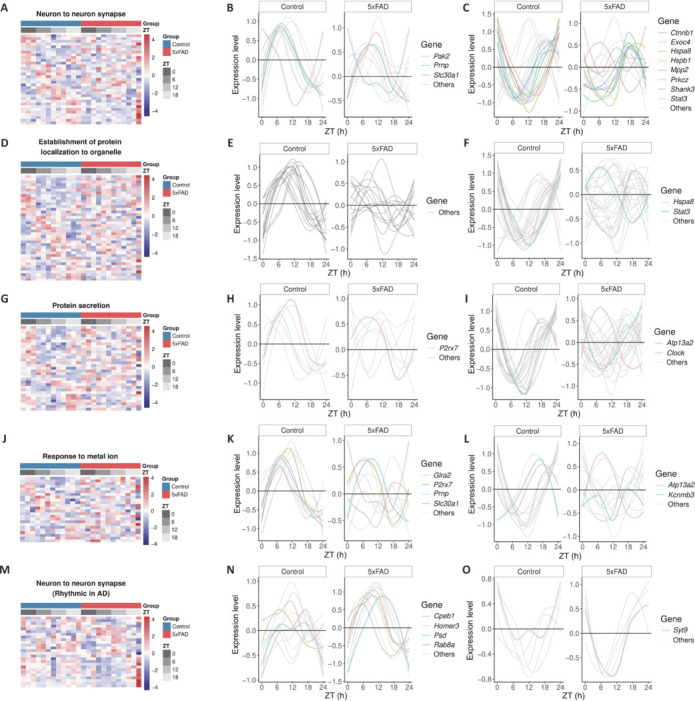
Hippocampal rhythmic genes lose their circadian expression patterns in Alzheimer’s disease (AD) mice. Heatmaps and fitted curve plots depicting the regulation of enriched pathway genes: (A–C) The neuron to neuron synapse (from I); (D–F) the establishment of protein localization to organelle (from I); (G–I) the protein secretion (from I); (J–L) the response to metal ion (from I); (M–O) the neuron to neuron synapse (from II). Each sample is represented by a column, and a line represents each gene. Each line in the fitted curve represents a gene; the expression of each gene is standardized, and the horizontal axis represents the ZT time. The curves colored show important genes mentioned in Figure 6. The data for ZT24 in the figure are the data for the replicated ZT0. AD: Alzheimer’s disease; Atp13a2: ATPase cation transporting 13A2; Cpeb1: cytoplasmic polyadenylation element binding protein 1; Ctnnb1: catenin beta 1; Exoc4: exocyst complex component 4; Glra2: glycine receptor Alpha 2; Homer3: homer scaffold protein 3; Hspa8: heat shock protein family A (Hsp70) member 8; Hspb1: heat shock protein family B (Small) member 1; Kcnmb3: potassium calcium-activated channel subfamily M regulatory beta subunit 3; Mpp2: MAGUK P55 scaffold protein 2; Npas2: neuronal PAS domain protein 2; P2rx7: purinergic receptor P2X 7; Pak2: P21 (RAC1) activated kinase 2; Prkcz: protein kinase C Zeta; Prnp: prion protein (Kanno blood group); Psd: pleckstrin and Sec7 domain containing; Rab8a: RAB8A, member RAS oncogene family; Shank3: SH3 and multiple ankyrin repeat domains 3; Slc30a1: solute carrier family 30 member 1; Stat3: signal transducer and activator of transcription 3; Syt9: synaptotagmin 9; ZT: zeitgeber.

To delve deeper into the circadian alterations of genes related to key biological functions in the hippocampus of 5-month-old AD mice, we selected genes from four pathways with the highest enrichment scores from the previous enrichment analysis: neuron-to-neuron synapse, establishment of protein localization to organelle, protein secretion, and response to metal ion. We compared gene expression between 5-month-old AD mice and age-matched controls (**Figure 5A–O**). For each pathway, we divided the rhythmic genes identified in the control hippocampus into two groups by whether their expression peaked between ZT0–12 or ZT12–24. In AD mice, we observed a loss of the synchronized oscillatory patterns present in control hippocampi (**Figure 5B**, **C**, **E**, **F**, **H**, **I**, **K**, and **L**).

**Figure 6:**
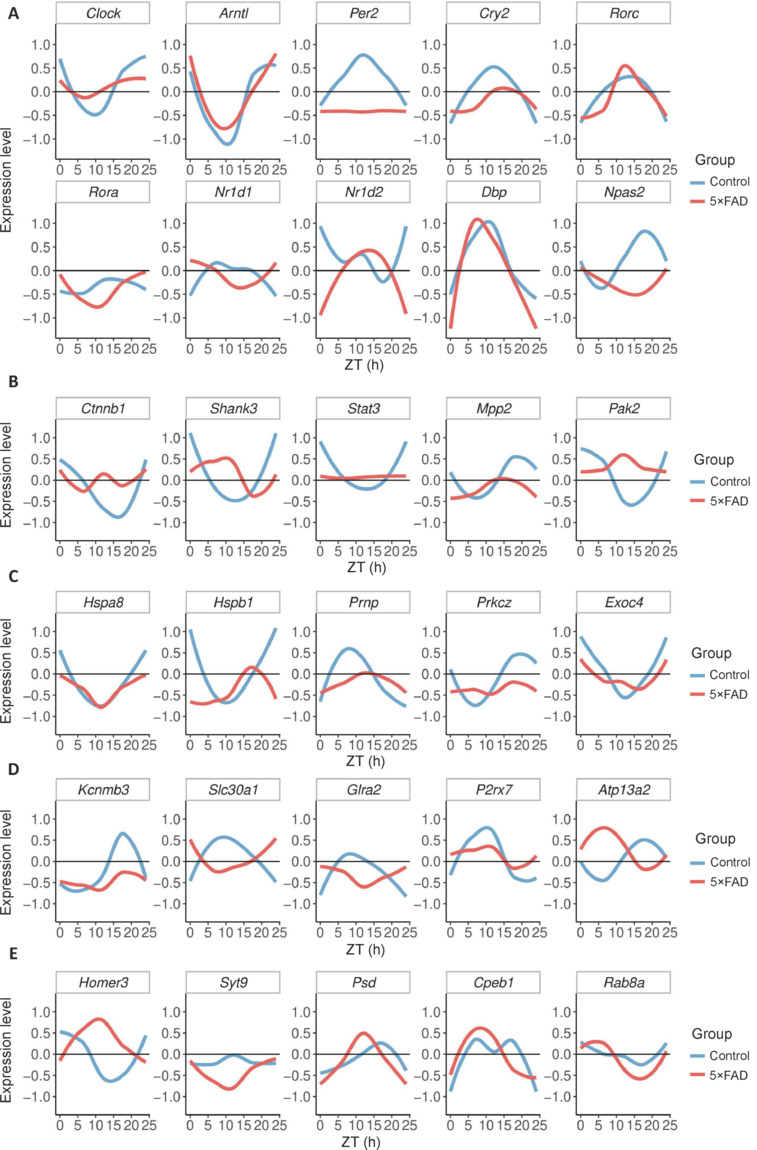
Disrupted circadian rhythms in genes involved in proteostasis, neuroinflammation, and ion homeostasis. (A–E) Diurnal expression of core clock genes (A); hippocampal neuronal function (B); protein quality control and degradation (C); ion channel related genes (D); rhythmic expression in AD mice (E). The expression of each gene is standardized, and the horizontal axis represents the ZT time. The data for ZT24 in the figure are the data for the replicated ZT0. Arntl: Aryl hydrocarbon receptor nuclear translocator like, or Bmal1; Atp13a2: ATPase cation transporting 13A2; Clock: clock circadian regulator; Cpeb1: cytoplasmic polyadenylation element binding protein 1; Cry2: cryptochrome circadian regulator 2; Ctnnb1: catenin beta 1; Dbp: D-box binding PAR BZIP transcription factor; Exoc4: exocyst complex component 4; Glra2: glycine receptor alpha 2; Homer3: homer scaffold protein 3; Hspa8: heat shock protein family A (Hsp70) member 8; Hspb1: heat shock protein family B (Small) member 1; Kcnmb3: potassium calcium-activated channel subfamily M regulatory beta subunit 3; Mpp2: MAGUK P55 scaffold protein 2; Npas2: neuronal PAS domain protein 2; Nr1d1: nuclear receptor subfamily 1 group D member 1; Nr1d2: nuclear receptor subfamily 1 Group D member 2; P2rx7: purinergic receptor P2X 7; Pak2: P21 (RAC1) activated kinase 2; Per2: period circadian regulator 2; Prkcz: protein kinase C zeta; Prnp: prion protein (Kanno blood group); Psd: pleckstrin and Sec7 domain containing; Rab8a: RAB8A, member RAS oncogene family; Rora: RAR related orphan receptor A; Rorc: RAR related orphan receptor C; Shank3: SH3 and multiple ankyrin repeat domains 3; Slc30a1: solute carrier family 30 member 1; Stat3: signal transducer and activator of transcription 3; Syt9: synaptotagmin 9.

We also examined a subset of genes in the neuron-to-neuron synapse pathway that were arrhythmic in control mice but gained circadian rhythmicity in the hippocampus of 5-month-old AD mice. Strikingly, genes that peaked between ZT0 and ZT12 in the AD hippocampus exhibited a day-high/night-low oscillatory pattern, whereas the same genes displayed no rhythmicity in age-matched controls (**Figure 5N** and **O**).

To explore the significance of these circadian alterations, we further analyzed the functional roles of these genes and their corresponding oscillatory patterns using CircaCompare to determine the significance of the difference. In summary, this analysis reveals that, in 5-month-old AD mice, hippocampal genes from four key pathways lose the synchronized oscillation seen in controls, while a subset of neuron-to-neuron synapse pathway genes gains circadian rhythm with a day-high/night-low pattern. These findings highlight pathway-specific circadian gene disruption in the AD hippocampus, which regulates the normal physiological activity of neurons in the hippocampus, and the altered expression patterns of these genes may affect Aβ clearance and neuroinflammation (Wang et al., 2025), and lay the groundwork for understanding how such alterations may contribute to AD pathogenesis.

### Disrupted circadian rhythms in genes involved in proteostasis, neuroinflammation, and ion homeostasis

To investigate whether AD affects the circadian regulation of genes in the hippocampus, we analyzed the 24-hour gene activity patterns in 5-month-old AD mice and control mice. We used CircaCompare (Parsons et al., 2020) to determine if the differences in these daily rhythms were significant (**Additional Table 3**). Core clock genes, such as *Clock*, *Per2*, and *Cry2*, exhibited notable changes in their daily expression levels in the AD mice (**Figure 6A**, *P* < 0.05). This indicates that circadian disruptions are occurring at the level of gene transcription.

**Additional Table 3:** Circacompare analysis results

Gene	*P*-value for amplitude difference	*P*-value for difference in phase	*P*-value for mesor difference	Presence of rhythmicity (*P*-value) for 5xFAD	Presence of rhythmicity (*P*-value) for Control
*Clock*	0.017	0.910	0.865	0.031	0.001
*Arnt1*	0.215	0.996	0.759	0.000	0.000
*Per2*	0.195	0.496	0.000	0.232	0.013
*Cry2*	0.713	0.045	0.004	0.050	0.007
*Rorc*	0.151	0.442	0.027	0.016	0.033
*Rora*	0.146	0.010	0.925	0.025	0.058
*Nrld1*	0.905	0.279	0.223	0.151	0.028
*Nr1d2*	0.283	0.025	0.633	0.003	0.041
*Dbp*	0.313	0.535	0.041	0.000	0.000
*Npas2*	0.822	0.761	0.994	0.277	0.018
*Ctnnb1*	0.031	0.486	0.623	0.109	0.000
*Rhank3*	0.064	0.247	0.050	0.556	0.001
*Stat3*	0.141	0.236	0.000	0.063	0.002
*Mpp2*	0.800	0.134	0.204	0.064	0.018
*Pak2*	0.868	0.005	0.000	0.089	0.001
*Hspa8*	0.402	0.008	0.816	0.369	0.000
*Hspbl*	0.022	0.000	0.617	0.164	0.000
*Prnp*	0.124	0.956	0.017	0.052	0.001
*Prkcz*	0.479	0.242	0.534	0.080	0.004
*Exoc4*	0.550	0.004	0.667	0.238	0.001
*Kcnmb3*	0.016	0.950	0.111	0.717	0.001
*Slc30a1*	0.042	0.002	0.704	0.066	0.000
*Glra2*	0.742	0.005	0.497	0.339	0.015
*P2rx7*	0.185	0.401	0.003	0.384	0.005
*Atp13a2*	0.712	0.000	0.155	0.157	0.014
*Homer3*	0.045	0.998	0.906	0.021	0.218
*Syt9*	0.446	0.405	0.322	0.025	0.198
*Psd*	0.318	0.690	0.000	0.000	0.180
*Cpeb1*	0.368	0.018	0.910	0.002	0.350
*Rab8a*	0.153	0.813	0.020	0.006	0.342

We also found numerous other genes involved in hippocampal neuron function that lost their daily rhythm or exhibited altered rhythms. Key synaptic genes such as Ctnnb1, Stat3, Shank3, Mpp2, and Pak2 exhibited significant disruptions in 24-hour expression levels in the AD mice (*P* < 0.05; **Figure 6B**). These disruptions included weaker rhythms (lower amplitude), shifted timing (phase shifts), or even a complete loss of their daily cycles. Additionally, genes associated with protein quality control and degradation processes—such as *Prnp*, *Prkcz*, *Exoc4*, and *Atp13a2*—showed major disturbances in daily rhythms (*P* < 0.05; **Figure 6C**). These changes may indicate that the cells are unable to maintain proper protein balance, potentially contributing to the early protein aggregation that is a hallmark of AD.

Membrane proteins, particularly ion channels, are crucial for the proper functioning of neurons because they directly control how neural signals are initiated, propagated, and modified. We examined how the circadian rhythms of genes encoding ion channels and their regulatory proteins change. We identified significant disruptions in the daily expression patterns of several key genes, including potassium channel *Kcnmb3*, the chloride channel *Glra2*, the ATP-gated cation channel *P2rx7*, the zinc transporter *Slc30a1*, and *Atp13a2*, which is involved in regulating the degradation of membrane proteins (*P* < 0.05; **Figure 6D**). These results indicate a widespread breakdown in the circadian control of genes encoding ion transport-related processes in the hippocampus of the AD mouse model.

Notably, we observed a set of genes that did not exhibit any circadian rhythm in age-matched healthy mice, but showed a pronounced daily pattern of expression in the hippocampus of 5-month-old AD mice (*P* < 0.05; **Figure 6E**). This is reminiscent of the changes that occur when stress disrupts the body’s circadian rhythms (Zhang et al., 2022b). These genes include *Homer3*, *Syt9*, *Psd*, *Cpeb1*, and *Rab8a*, all of which encode proteins that play roles in synaptic signaling, local protein production, or vesicle transport. In the AD mice, *Homer3* exhibited a distinct pattern, with high levels during the day and low levels at night. *Syt9* and *Rab8a* also exhibited a daily rhythm, aligning with time-of-day changes in neurotransmission and vesicle trafficking. *Cpeb1* and *Psd* also displayed daily fluctuations. This suggests that, under pathological conditions, there is a regulated timing of synaptic protein production and structural maintenance. The emergence of a daily rhythm in these synaptic genes may represent the brain’s attempt to adapt to early synaptic dysfunctions in AD. This may serve as a compensatory mechanism to support neuronal communication and maintain balance within the brain.

## Discussion

### Circadian rhythm disruptions emerge before cognitive symptoms start in the mouse model of Alzheimer’s disease

In this study, we found that disruptions in circadian rhythms occur before cognitive symptoms manifest in AD model mice. To investigate the changes in hippocampal gene expression in AD, we conducted high-resolution 24-hour transcriptomic profiling of the hippocampus in 5-month-old AD model mice, a time point preceding any apparent cognitive decline. Our data reveal widespread disruption of circadian gene expression rhythms in the hippocampus, occurring even before behavioral symptoms appear. Notably, most of the rhythmic genes that are dysregulated are involved in neuronal function, protein balance, and immune regulation. These findings suggest that circadian dysfunction is not merely a side effect of neurodegeneration and rather, it may represent an early, inherent pathological feature of AD. The early disruption of rhythmic patterns in the hippocampus may render this brain region more susceptible to synaptic failure, protein aggregation, and neuroinflammation. Therefore, circadian dysfunction may serve as a prodromal biomarker and a potential target for early intervention in AD.

It is worth highlighting that many of the genes with disrupted daily rhythms in AD model mice are involved in pathways such as synaptic signaling, plasticity, and protein folding. This underscores the importance of daily regulation of genes for maintaining the health of the hippocampus. For example, *Ctnnb1* encodes β-catenin, a key component of the Wnt signaling pathway, which is crucial for neuronal development, survival, and synapse maintenance (Li et al., 2008; Maguschak and Ressler, 2008; Liu et al., 2022). Normally, the expression level of *Ctnnb1* exhibits a 24-hour cycle of activity, but in AD mice, the cycle completely disappears. Stat3 encodes a transcription factor that helps protect neurons and regulate inflammation (Traxler et al., 2022). In AD mice, its levels are significantly lower, and it loses its typical pattern of being high during the day and low at night. *Shank3* encodes a critical scaffold protein at the postsynaptic side of excitatory synapses (Cai et al., 2021). Normally, its expression reaches the lowest point at ZT2, but this pattern is absent in AD mice. Even genes involved in maintaining synaptic integrity and organizing the cell’s cytoskeleton exhibit disrupted daily rhythms (Wang et al., 2018). *Pak2*, in particular, shows much lower levels and even has reversed peak and trough timings in AD model mice. This suggests that the early disruption of the daily rhythms of these genes, which are involved in neuronal signaling and synaptic structure, may significantly contribute to the hippocampal dysfunction observed in AD.

When genes involved in proteostasis lose their daily rhythms, this may lead to exacerbated neurodegeneration by disrupting protein folding and clearance. Misfolded Aβ and tau proteins are major contributors to AD pathology, as they disrupt synapses and play a central role in the disease (Knopman et al., 2021). *Hspa8* (also known as *Hsc70*) encodes a molecular chaperone that helps clear out Aβ and manage misfolded proteins (Xu et al., 2021). In our study, both its overall levels and its 24-hour rhythm decreased significantly in AD model mice. Similarly, *Hspb1*, which encodes a protein that maintains protein balance by working with autophagy and the ubiquitin-proteasome system (Mymrikov et al., 2011), also lost its rhythmic expression pattern. Our data showed that the daily fluctuations of *Prnp* weakened considerably. The direct role of *Prnp* in AD is not yet clear, but it has been linked to neuroprotection and the management of misfolded proteins in other neurodegenerative diseases (Zerr et al., 2024). Other genes, such as *Prkcz*, which encodes a regulator of protein breakdown, and *Exoc4*, which encodes a protein involved in moving proteins via vesicles (Hsu et al., 1999; Malik et al., 2022), also exhibited disrupted daily rhythms. This suggests that in early AD, the daily regulation of protein quality control systems breaks down, which may lead to protein imbalances and increased vulnerability of neurons.

Membrane proteins, particularly ion channels, are essential for neuronal signaling as they directly control the initiation, propagation, and modulation of action potentials. In this study, we profiled the circadian expression of ion channel-related genes in the hippocampus and found extensive rhythm disruption in the AD model. *Kcnmb3*, which encodes a β-subunit of the large-conductance calcium-activated potassium channel (Uebele et al., 2000), displayed a robust peak from ZT12 to ZT24 in wild-type mice, but this peak was lost in AD mice. This arrhythmic expression may impair circadian K^+^ regulation, potentially contributing to synaptic hyperexcitability. *Glra2*, which encodes a glycine receptor Cl⁻ channel involved in inhibitory transmission (Zhang et al., 2015), was significantly downregulated in AD mice and exhibited a phase reversal in its rhythmic pattern. Such changes could lead to a loss of nighttime inhibitory control, increasing network excitability and impairing memory consolidation. *P2rx7*, which encodes an ATP-gated ion channel enriched in microglia, was markedly upregulated, consistent with its role in neuroinflammation via IL-1β release (Huang et al., 2024). Notably, previous studies have shown that pharmacological inhibition of P2X7 receptors, using agents such as Brilliant Blue G or GSK1482160, attenuated neuroinflammatory responses and reduced amyloid burden in AD models, suggesting that P2rx7 serves not only as a biomarker but also as a potential therapeutic target (Martin et al., 2019; Ronning et al., 2023). *Slc30a1*, which encodes the zinc transporter ZnT1 (Li et al., 2024), lost its normal day-high/night-low rhythmicity, suggesting impaired temporal zinc homeostasis that could disrupt synaptic function and enhance Aβ deposition. *Atp13a2*, which encodes a P-type ATPase involved in metal ion regulation and autophagy (van Veen et al., 2020), exhibited an inverted circadian pattern in AD mice, resulting in potentially weakened waste clearance during periods of high demand. These disruptions in the rhythms of ion channel and transporter genes may contribute to ion imbalances, impaired proteostasis, and progressive neurodegeneration.

We also identified de novo rhythmicity in several genes not previously known to oscillate in the hippocampus, including *Homer3*, *Syt9*, *Cpeb1*, and *Rab8a*. These genes encode proteins involved in synaptic plasticity, vesicle release, mRNA translation, and membrane trafficking, and their rhythmic emergence in AD mice may reflect compensatory adaptations to heightened synaptic or proteostatic stress (Xiao et al., 2021; Seibert et al., 2023; Singh et al., 2024). The functional significance of the gains in rhythmicity remains to be fully determined.

Previous studies have discussed circadian disruptions in late-stage AD models and patients. Disruptions to the SCN regulated circadian rhythm system impair the rhythmicity of key physiological processes, including microglial phagocytic activity and synaptic plasticity. Since microglia possess an intrinsic circadian rhythm regulated by clock genes, disruptions to this rhythm impair their ability to clear Aβ aggregates and regulate neuroinflammation, thereby promoting Aβ accumulation and a neuroinflammatory cascade that drives early neurodegeneration (Geng et al., 2025). As AD progresses, circadian rhythm disruption becomes a significant secondary manifestation: neurodegeneration of the SCN, cholinergic defects, and widespread tau protein lesions directly disrupt the central circadian rhythm pacemaker, exacerbating sleep-wake cycle disturbances, diurnal mood fluctuations, and cognitive impairment (Rigat et al., 2023). This further exacerbates the pathological process of AD. Sleep disturbances reduce the brain’s lymphatic system’s clearance of toxic proteins, while persistent circadian rhythm disorders exacerbate neuroinflammation, thus forming a self-reinforcing cycle that accelerates disease progression (Nassan and Videnovic). However, our findings indicate that molecular circadian alterations begin before cognitive issues even appear. This supports the idea that circadian dysfunction is not merely a result of neurodegeneration—it might actually be an upstream driver. One possible reason for these molecular circadian alterations is that the core clock genes in hippocampal neurons or glial cells cease oscillating properly. Another possibility is that the body’s overall circadian signals, perhaps from the suprachiasmatic nucleus (SCN), become disrupted early in the disease. Future research using cell type-specific tracking tools or circadian interventions, such as time-restricted feeding or light therapy, may help clarify whether circadian regulation directly causes AD-related changes.

Most previous studies sampled single time points from sparsely profiled cortex or hippocampus to study pathological changes (Liu et al., 2021) and molecular pathways and physiological changes (Wang et al., 2025), risking the potential to miss oscillations and phase shifts. Here, we present a dense 24-hour hippocampus-resolved transcriptomic atlas at a pre-symptomatic stage. Our results in AD model mice demonstrate that circadian dysregulation of gene expression, including loss of rhythmicity, phase inversion, and amplitude dampening, precedes cognitive decline. Disruptions clustered into modules spanning synaptic signaling (*Shank3*, *Mpp2*, *Pak2*), proteostasis (*Hspa8*, *Hspb1*, *Prkcz*, *Exoc4*), and metal ion homeostasis (*Kcnmb3*, *Glra2*, *P2rx7*, *Slc30a1*, *Atp13a2*), positioning the hippocampal clock as a systems-level regulator of neuronal homeostasis. We also identified de novo rhythmicity of a subset of genes (*Homer3*, *Syt9*, *Cpeb1*, *Rab8a*), consistent with compensatory re-timing, which is a unique finding in a field that has largely focused on uniform rhythm loss.

Recent studies have shown that correcting circadian rhythm disruptions helps slow the progression of AD. Research has found that time-restricted feeding can regulate genes related to neuroinflammation and reduce the pathological changes associated with AD (Whittaker et al., 2023). A previous study demonstrated that morning light therapy effectively improves circadian rhythm disorders and cognitive deficits in patients with AD (Canazei et al., 2022). These therapeutic effects may stem from the strong synchronization effects of light on the SCN master clock (Hoyt and Obrietan, 2022). Stabilizing the synchronization of the SCN clock ensures that the core clock maintains the correct phase relationship with the 24-hour cycle. Furthermore, existing studies have proposed evidence of circadian disruption in patients with Alzheimer’s disease in the preclinical stage, but there is a lack of data showing whether pre-symptomatic circadian disruption is linked to later pathological progression (Zhao et al., 2025). This information is essential to confirm that circadian markers can be used as early biomarkers for AD.

## Limitations

This study relied on a single mouse model of AD, which may not fully capture the genetic and pathological heterogeneity inherent to human AD. Additionally, our transcriptomic data only reflect gene expression patterns. While the observed disruptions in rhythmic gene expression are significant, whether these changes translate to alterations in protein abundance or function, both of which are critical for physiological impact, remains unknown. Furthermore, the study focused exclusively on the hippocampus and did not investigate circadian rhythms in other brain regions relevant to AD. Disruptions in these regions might interact with hippocampal dysrhythmia to drive AD progression, and more research is needed to obtain a system-level understanding of circadian dysfunction in AD.

Another limitation is the absence of *in vivo* validation for the functional consequences of the identified rhythm disruptions. Such experiments are necessary to confirm whether circadian disruption acts as a causal factor in hippocampal impairment rather than merely a correlative one. Finally, the study lacks human data to determine whether pre-symptomatic AD patients exhibit similar hippocampal circadian gene dysregulation. Addressing these questions will require analyzing rhythmic gene expression in post-mortem hippocampal tissue from pre-symptomatic individuals or developing non-invasive tools to assess hippocampal rhythms.

## Conclusion and outlook

Our results demonstrate that circadian rhythm disruption is an early and widespread molecular feature in the hippocampus of AD model mice. This disruption affects genes that are key to neuronal communication, synaptic stability, protein balance, and immune function. Targeting circadian mechanisms may be a promising approach for early intervention and alter the course of AD. Additionally, the emergence of new rhythmic patterns in genes related to synaptic plasticity suggests potential compensatory mechanisms, which should be further explored as therapeutic targets. Future research should focus on understanding the molecular mechanisms behind circadian disruptions in AD and exploring circadian-based therapies, such as light therapy or time-restricted feeding, to restore circadian balance. These approaches may represent key strategies for slowing AD progression, particularly when implemented during the pre-symptomatic stages.

## Additional files:

***Additional Table 1:*** *Real-time PCR primer sequences.*

***Additional Table 2:*** *GO enrichment analysis results.*

***Additional Table 3:*** *Circacompare analysis results.*

## Data Availability

*All relevant data are within the paper and its Additional files.*.
